# Body mass index and prevalence of metabolic syndrome among Korean adults before and after the COVID-19 outbreak: a retrospective longitudinal study

**DOI:** 10.4178/epih.e2023081

**Published:** 2023-08-29

**Authors:** Joo-Eun Jeong, Hoon-Ki Park, Hwan-Sik Hwang, Kye-Yeung Park, Myoung-Hye Lee, Seon-Hi Shin, Nayeon Choi

**Affiliations:** 1 Department of Family Medicine, Hanyang University College of Medicine, Seoul, Korea, Seongdong-gu, Korea; 2 Biostatistical Consulting and Research Lab, Medical Research Collaborating Center, Hanyang University, Seoul, Korea

**Keywords:** COVID-19, Pandemics, Metabolic syndrome, Body mass index, Public health

## Abstract

**OBJECTIVES:**

Studies evaluating weight changes during the coronavirus disease 2019 (COVID-19) pandemic have yielded inconsistent results, and most of those studies were based on self-reported anthropometric measures. We investigated changes in body mass index (BMI), professionally measured waist circumference (WC), and metabolic syndrome components from before to during the pandemic in a sample of the adult population in Korea.

**METHODS:**

This retrospective study included 1,118 male and female (age≥18 years) who underwent health checkups at a university medical center between January 1, 2016 and March 31, 2022. Changes in BMI, lifestyles, and metabolic syndrome components during the pandemic were analyzed using the paired t-test, McNemar test, generalized estimating equations, and repeated-measures analysis of variance.

**RESULTS:**

Changes in body weight, BMI, and body fat percentage during the pandemic were not clinically significant. However, statistically significant results were found for decreased physical activity (p<0.001) and WC (p<0.001), and exacerbation of all metabolic syndrome components (except serum triglyceride levels). Moreover, the metabolic syndrome prevalence increased significantly from 20.2% to 31.2% during the pandemic (p<0.001). The prevalence of abdominal obesity and high fasting blood glucose levels also significantly increased from 2019 to 2021.

**CONCLUSIONS:**

Metabolic syndrome, its components, and fat distribution worsened significantly after the implementation of social distancing and lockdowns, despite no clinically significant changes in body weight and BMI. Further studies on the post- pandemic period should investigate the long-term impact of social lockdowns on BMI and the prevalence of metabolic syndrome.

## GRAPHICAL ABSTRACT

[Fig f6-epih-45-e2023081]

## INTRODUCTION

Coronavirus disease 2019 (COVID-19), caused by severe acute respiratory syndrome coronavirus 2 (SARS-CoV-2) [1] was declared a global pandemic by the World Health Organization (WHO) on March 11, 2020 [2]. In response, numerous countries enacted control measures such as social distancing and lockdowns, advising or requiring people to remain at home and work remotely. Despite intensive efforts to contain the spread of COVID-19, the global tally of confirmed cumulative cases and deaths had surpassed 660 million and 6 million, respectively, by January 2023 [3].

The Korean government first implemented a social distancing system on March 22, 2020 [4]. This system restricted the use of public facilities, such as churches, temples, schools, clubs, and gyms. In November 2021, a new policy known as the “vaccine pass” was introduced. This pass served as an official record of an individual’s COVID-19 vaccination status, which was required for access to public facilities. Those who were not fully vaccinated had to present a negative COVID-19 polymerase chain reaction test result or a negative COVID-19 antigen test result obtained within the previous 48 hours. Failure to comply resulted in restricted access to a wide range of public facilities [5]. The pass was discontinued on March 1, 2022, because the Korean government decided to gradually ease COVID-19 health protocols due to the predominance of the Omicron variant, which is more contagious but less severe than the former Delta variant [6].

Social distancing was a necessary and unprecedented policy that prevented the spread of the highly contagious SARS-CoV-2. However, it also fundamentally transformed people’s lifestyles, affecting their health and behaviors alike [7,8]. Restrictions on outdoor activities forced people to stay at home for longer periods and increased their sedentary time [9]. Dietary behaviors also deteriorated, with increased intake of snacks, fast food, and delivered food [10,11]. Mental health was also negatively impacted, as the prolonged lockdowns exacerbated anxiety, depression, and stress about an uncertain future [12]. The deterioration of mental health negatively affected physical activity and eating behaviors, thereby worsening the vicious cycle [13].

Numerous studies have established the impact of COVID-19-related social distancing on weight gain and deterioration of metabolic markers [14-16]. Although several studies have revealed significant weight gain, some have yielded contradictory results [17,18]. Furthermore, most recent studies on the impact of COVID-19-related social distancing and lockdowns were based on self-reported questionnaires, which may lack reliability and accuracy [15,16]. In addition, these studies mostly focused on the year 2020, which may not accurately reflect longer-term metabolic and behavioral changes. Many previous studies have shown that obesity is a major risk factor for metabolic syndrome [19,20]. It increases the risk of comorbidities, such as type 2 diabetes mellitus, cardiovascular disease, and cerebrovascular disease [21]. Moreover, obesity may increase an individual’s vulnerability to COVID-19-related hospitalization and mortality [22].

While social distancing measures and lockdowns may curb the spread of COVID-19, they could have adverse effects on health, particularly in relation to weight and metabolism. As discussed above, previous studies on weight changes during the pandemic have yielded inconsistent results. Furthermore, these studies utilized data from 2020 and earlier, which may not fully capture the effects of the COVID-19 lockdown period. To the best of our knowledge, this is the first Korean study to examine both metabolic and behavioral changes, incorporating data from 2022. Consequently, our research aims to explore: (1) changes in body mass index (BMI) and the prevalence of metabolic syndrome (and its components) before and after the COVID-19 outbreak, up until the termination of the vaccine pass policy in March 2022, and (2) the influence of social distancing measures and lockdowns on lifestyle modifications.

## MATERIALS AND METHODS

### Study design and population

**Figure 1.**
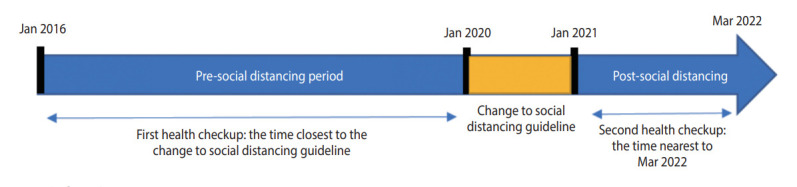
Study flow diagram.

This retrospective, longitudinal study utilized health checkup data from a university hospital in Seoul, Korea. The inclusion criteria were as follows: ≥ 18 years, availability of health-checkup data from 2016, and availability of health-checkup data from at least 1 follow-up conducted between January 1, 2021 and March 31, 2022. Data from 2020 were not included initially, but were later included in a sub-cohort analysis to ensure a sufficient follow-up period to reflect metabolic and behavioral changes. The study population was divided into the pre-pandemic period (checkups performed between January 1, 2016 and December 30, 2019) and the post-pandemic period (checkups performed between January 1, 2021 and March 31, 2022). If a participant underwent multiple checkups during the pre-pandemic period, the health checkup data obtained at the time closest to the transition to systematic social distancing were included in the total cohort. If a participant underwent multiple checkups during the pandemic, the data obtained nearest to March 2022 were included in the total cohort (Figure 1).

**Figure 2.**
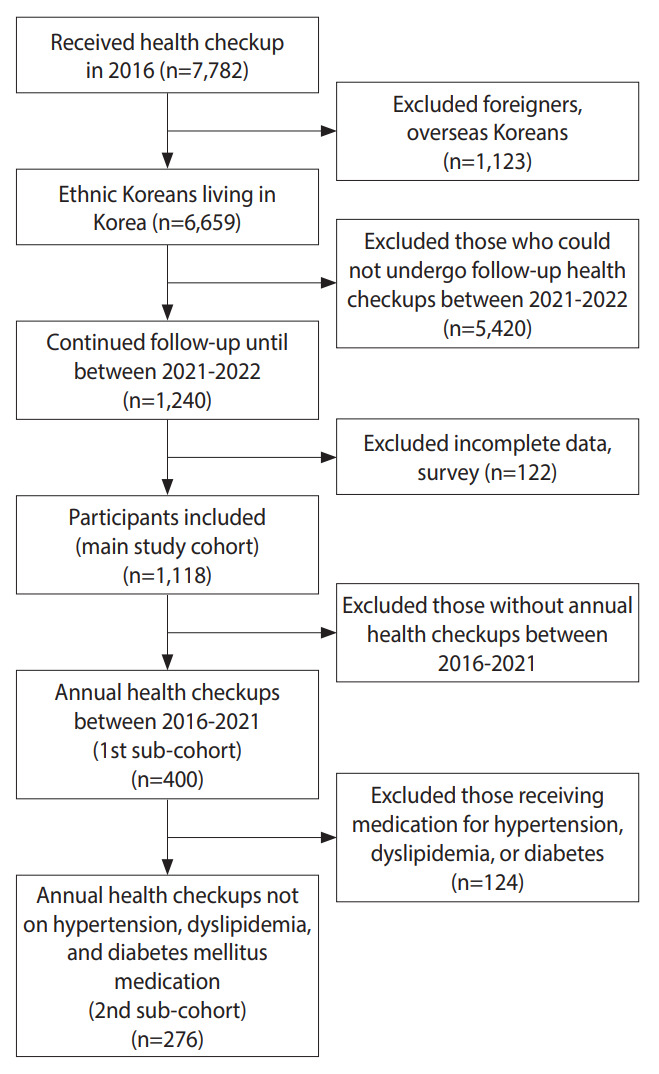
Flow chart of the study population.

The exclusion criteria were as follows: foreigners; overseas Koreans; individuals without follow-up checkups between 2021 and 2022; and individuals with incomplete data or questionnaire responses on underlying diseases, physical activity, and alcohol consumption. These criteria were confirmed to have been met by reviewing self-recorded questionnaires, medical records, anthropometric measurements, and laboratory test results. Overall, 1,118 participants were included in the initial dataset. Among these, data from 400 participants who underwent annual health checkups between 2019 and 2021 were included in the first sub-cohort. Among the 276 participants who were not taking any medications for underlying hypertension, dyslipidemia, and type 2 diabetes mellitus were included in the second sub-cohort (Figure 2). The sub-cohort analyses aimed to investigate whether there were meaningful changes throughout the COVID-19 pandemic and the pattern each change exhibited, while minimizing maturation bias and medication effects.

### Measurements and definitions

The participants visited the health checkup center after overnight fasting. Height was measured to the nearest 0.1 cm in the standing position using a stadiometer. Body weight was measured with the participants wearing light clothing and standing barefoot on digital scales. Waist circumference (WC) was measured to the nearest 0.1 cm using a tape measure; the anatomical site used for measurement was the midpoint between the lower margin of the rib cage and the highest point of the iliac crest. BMI was calculated using the following equation: weight (kg)/height (m2). Body fat percentage was measured using bioelectrical impedance analysis (Inbody 770; Inbody Co., Ltd, Seoul, Korea).

Blood samples for blood glucose and serum lipid measurements were collected after overnight fasting. Participants with self-reported diabetes mellitus and those taking anti-diabetic medications were defined as having diabetes mellitus. Participants with self-reported hypertension and those taking anti-hypertensive agents were defined as having hypertension. Finally, participants with self-reported dyslipidemia and those taking lipid-lowering agents were defined as having dyslipidemia.

A diagnosis of metabolic syndrome was established if at least 3 of the following conditions were met (according to the Third Report of the Adult Treatment Panel definition): (1) WC≥ 90 cm for male and ≥ 85 cm for female (using the modified WC criteria for abdominal obesity proposed by the Korean Society for the Study of Obesity [23]), (2) serum triglyceride (TG) level ≥ 150 mg/dL or use of lipid-lowering medications, (3) high-density lipoprotein cholesterol (HDL-C) level < 40 mg/dL for male and < 50 mg/dL for female, or use of lipid-lowering medications; (4) systolic blood pressure (BP) ≥ 130 mmHg, diastolic BP ≥ 85 mmHg, or use of anti-hypertensive drugs; and (5) fasting blood glucose (FBG) level ≥ 100 mg/dL or use of anti-diabetic medications.

Using a self-reported questionnaire, we conducted a survey on the following lifestyle parameters: smoking status, medical history, alcohol consumption, and extent of exercise. The survey inquired about the average number of cigarettes smoked daily, the duration of smoking in years, the frequency of alcohol consumption per week, the quantity of alcohol consumed in a single sitting (measured in drinks), and the types of alcohol consumed. We calculated weekly alcohol consumption using the following formula: drink volume (mL) × number of drinks × frequency of drinking per week× alcohol concentration by volume (%)× specific gravity of alcohol (0.79) [24]. We used the Korean version of the International Physical Activity Questionnaire short form to determine the number and duration of regular exercise sessions performed at high-intensity, intermediate, and walking levels each week. We calculated the metabolic equivalent of task (MET) by multiplying the given MET value (high-intensity: 8, intermediate: 4, and walking: 3.3) by the minutes of activity per week [25].

### Statistical analysis

Baseline characteristics are presented as mean± standard deviation (SD) or as number (%). Continuous and categorical variables among the baseline characteristics were compared between the pre-pandemic and during-pandemic groups using the paired t-test and the McNemar test, respectively. Measures of effect size were calculated using Cohen’s d when the paired t-test was conducted. For the paired contingency table analysis when the McNemar test was performed, Cohen’s g was computed. Metabolic parameters were compared between the pre-pandemic and during-pandemic cohorts using the McNemar test. Furthermore, 2 sub-cohorts were created to observe acute changes during the period of transition to social distancing and to minimize maturation bias and underlying medication effects. Annual health checkup data from the first and second sub-cohorts were analyzed using generalized estimating equations and repeated-measures analysis of variance, respectively. All analyses were performed using SAS version 9.4 (SAS Institute Inc., Cary, NC, USA). Statistical significance was set at p<0.05.

### Ethics statement

This study was approved by the Ethics Committee of the Hanyang University Seoul Hospital (approval No. 2023-03-017) and conducted in accordance with the Declaration of Helsinki. The patient consent requirement was waived because this was a retrospective longitudinal cohort study based on an anonymized database provided by the health authorities.

## RESULTS

### Comparison of participant characteristics before and during the pandemic

Table 1 presents a comparison of the pre-pandemic and during-pandemic baseline characteristics. The mean follow-up period was 2.3 years. During the pandemic, the prevalence rates of hypertension, dyslipidemia, and diabetes mellitus increased from 17.5%, 8.4%, 5.1% to 22.6%, 15.0%, and 7.7%, respectively (p<0.001 for all). The effect sizes of these 3 binary variables were extremely large. In other words, the increases in hypertension, dyslipidemia, and diabetes were remarkable. Conversely, the smoking rate decreased from 19.6% to 15.5% (p<0.001). The average weekly alcohol consumption increased from 78.5 g to 81.8 g during the pandemic; however, this difference was not statistically significant. Conversely, physical activity decreased from 1,831.4 to 1,500.1 MET× minutes during the pandemic (p<0.001). While the change in the average weight from before to during the pandemic was statistically significant, it was not considered clinically significant; moreover, the average BMI did not change significantly. However, the body fat percentage, total cholesterol level, and low-density lipoprotein cholesterol (LDL-C) levels increased significantly during the pandemic (p<0.001). All metabolic syndrome components, except for serum TG levels, were significantly exacerbated.

### Changes in the prevalence of metabolic syndrome and its components from before to during the pandemic

**Figure 3.**
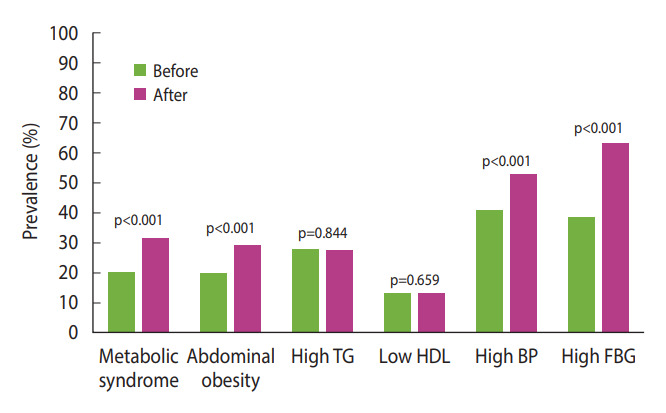
Changes in the prevalence of metabolic syndrome and its components from before to after the pandemic (n=1,118). TG, triglyceride; HDL, high-density lipoprotein; BP, blood pressure; FBG, fasting blood glucose.

The prevalence of metabolic syndrome and its components was compared before and during the pandemic (Figure 3). The overall prevalence of metabolic syndrome increased from 20.2% to 31.2% during the pandemic (p<0.001). The proportion of participants in whom WC increased from normal to indicative of abdominal obesity was 9.5% (p<0.001). The proportions of patients with high serum TG levels (≥150 mg/dL) and those with low HDL-C levels increased by 0.4% and 0.5%, respectively; however, these increases were not statistically significant. The proportion of patients with high BP increased by 12% (p<0.001). Finally, the proportion of patients with high FBG levels (≥ 100 mg/dL) increased by 24.9% (p<0.001); this was the largest increase among all changes in the metabolic syndrome components.

### Subgroup analyses

In the 2 sub-cohort analyses, we investigated the significant differences between the sex groups in longitudinal trends. More specifically, the first sub-cohort (n_1_= 400) had records of annual health checkups between 2016 and 2021, and the second sub-cohort (n_2_= 276) was the remainder, excluding those who received medical treatment for hypertension, dyslipidemia, or diabetes within the first sub-cohort. To investigate changes after social distancing was implemented, the study period was limited to 2019-2021.

### Trends in the prevalence of metabolic syndrome and its components from 2019 to 2021 among participants with annual health checkups (n_1_=400)

**Figure 4.**
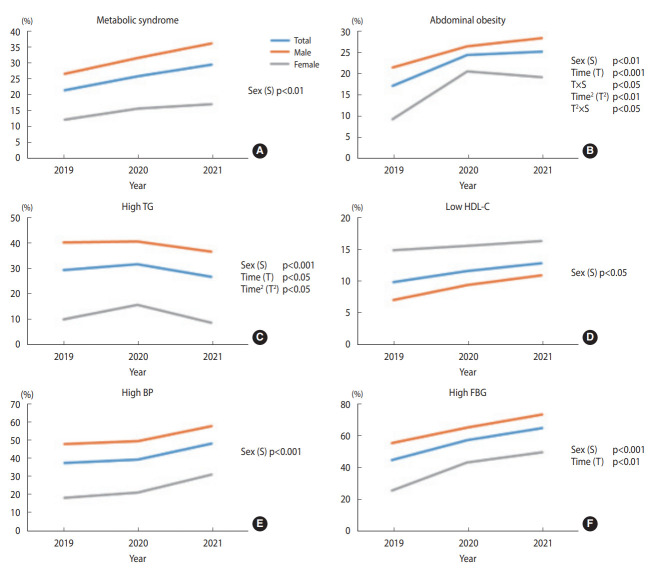
Trends in the prevalence of metabolic syndrome (A) and its components (B) abdominal obesity, (C) high triglyceride (TG), (D) low high density lipoprotein-cholesterol (HDL-C), (E) high blood pressure (BP), and (F) high fasting blood glucose (FBG) from 2019 to 2021 among participants with annual health checkups (n_1_=400). The p-values are from the generalized estimating equations approach.

Figure 4 shows the prevalence of metabolic syndrome and its components in the first sub-cohort. Supplementary Material 1 shows the results with significant p-values. Each prevalence plot shows the proportion of the disease at each time point, corresponding to each year. The prevalence of abdominal obesity increased significantly in a linear and quadratic manner over the study period. However, the sex groups differed significantly in these trends; females linear and quadratic trends appeared more salient than their counterparts. High TG decreased linearly and in a quadratic manner, while the quadratic trends were more salient than the linear trends in both sex groups. High FBG linearly increased for both males and females. The remaining characteristics, such as metabolic syndrome, low HDL, and high BP, showed no significant linear or quadratic changes.

### Changes in metabolic syndrome components from 2019 to 2021 among participants not taking anti-hypertensive, anti-diabetic, and lipid-lowering agents (n_2_=276)

**Figure 5.**
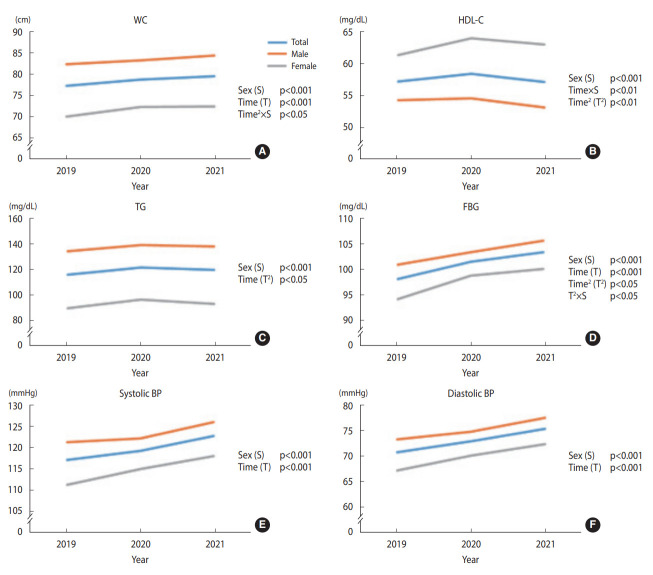
Changes in metabolic syndrome components (A) waist circumference (WC), (B) high density lipoprotein-cholesterol (HDL-C), (C) triglyceride (TG), (D) fasting blood glucose (FBG), (E) systolic blood pressure (BP), and (F) diastolic BP from 2019 to 2021 among participants not taking antihypertensive, anti-diabetic, and lipid-lowering agents (n_2_=276). The p-values are from the general linear model (analysis of variance with repeated measures).

Figure 5 shows the changes in metabolic syndrome components from 2019 to 2021 in the second sub-cohort. Supplementary Material 2 shows the significant p-values. WC demonstrated a significant linear trend over time, regardless of sex. In addition, the quadratic trend significantly differed between the sex groups; females WC increased noticeably between 2019 and 2020 and plateaued between 2020 and 2021, unlike male. The HDL-C showed a significant quadratic trend over time for both sex groups. However, the linear trend significantly differed between groups, as indicated by a significant time-and-group interaction effect (p<0.01). Females HDL-C increased over the research period, whereas males overall HDL-C level decreased. Both sex groups demonstrated a significant quadratic trend in TG, while the female group exhibited the trend more noticeably. FBG demonstrated a linearly increasing trend over time for both sex groups. However, the quadratic trend differed significantly between the groups; females inflection points stood out more than males. The overall FBG levels showed a greater increase between 2019 and 2020 than between 2020 and 2021 (p=0.013). Both systolic BP and diastolic BP linearly increased over time, regardless of sex.

## DISCUSSION

We investigated changes in the BMI, lifestyles, and prevalence of metabolic syndrome from before to after the implementation of social distancing norms and lockdowns during the COVID-19 pandemic using health checkup data collected at a university hospital between January 2016 and March 2022. The prevalence of hypertension, dyslipidemia, diabetes mellitus, and metabolic syndrome increased significantly (despite no clinical changes in body weight) after the implementation of the high-level social distancing measures. Regarding lifestyle habits, the smoking rate and amount of physical activity per week decreased significantly, whereas weekly alcohol consumption increased without statistical significance.

To observe general trends in the prevalence of metabolic syndrome and to elucidate the impact of the COVID-19 pandemic, we broadened the range of participants to include those who underwent checkups in 2016. Among these participants, we selected those with data from at least 1 follow-up checkup performed between January 1, 2021 and March 31, 2022. To ensure a sufficient follow-up period for acute changes to occur, we did not include data from 2020 (i.e., the year when WHO declared COVID-19 a global pandemic).

We found no clinically significant changes in body weight, BMI, and body fat percentage, but noted a significant increase in the prevalence of abdominal obesity during social distancing. This finding is contradictory to the findings of previous studies that reported prominent weight gain during the pandemic and associated social distancing measures and/or lockdowns [13,15,16]. This discrepancy may be explained as follows. First, as mentioned earlier, previous studies based their analyses on questionnaire responses rather than measured data, which are subject to recall bias. Conversely, we obtained our data from direct anthropometric and body composition measurements. Second, our study design and follow-up period duration differed from those of previous studies. We used a retrospective longitudinal design with a broad follow-up period and paired data. Notably, our results are consistent with those of 2 previous longitudinal studies [17,18].

Although the BMI did not increase significantly, the significant increase in WC indicates adverse changes in the metabolic status [19]. Most metabolic parameters, except for serum TG levels, were exacerbated. We suggest that the main factors underlying this outcome are lifestyle changes caused by the COVID-19 pandemic and related social distancing measures. Although data on the participants’ eating habits were lacking, our data revealed a significant decrease in their physical activity. The average alcohol consumption increased, but this increase was not statistically significant. Previous studies have revealed lifestyle changes during the COVID-19 pandemic, such as an increase in the consumption of poor-quality foods [10,11]. Restrictions to suppress SARS-CoV-2 transmission also led to a decrease in physical activity [9]. Furthermore, some studies have reported increased alcohol consumption during the initial phase of the pandemic [26]. However, several studies have also found a decrease in alcohol consumption since the implementation of social distancing measures in Korea [27]. In Korea, the intensity of social distancing measures was adjusted according to the regional severity of COVID-19 [28]; this may explain our statistically insignificant findings regarding alcohol consumption.

Total cholesterol, HDL-C, and LDL-C levels increased during the social distancing period. FBG levels also markedly increased, which may be related to the exacerbation of central obesity and fat distribution, reflecting the worsening of insulin resistance due to decreased physical activity [29].

The 2 subgroup analyses enabled us to not only investigate acute changes during the transitional period to social distancing but also minimize bias and the effects of anti-hypertensive, anti-diabetic, and lipid-lowering agents. During 2019 and 2021, a linear increase in the prevalence of metabolic syndrome components (such as abdominal obesity and high TG and FBG levels) was observed. WC, FBG levels, and systolic BP and diastolic BP increased linearly during the pandemic, while HDL-C and TG levels changed in 2020. These findings confirm the exacerbation of fat distribution despite the lack of a significant change in BMI. The worsening of FBG levels was the most prominent change among the changes in the metabolic syndrome components; it implied an adverse change in insulin resistance during the social distancing period. Females were more affected than males, as the prevalence of abdominal obesity, WC, and HDL-C and FBG levels showed steeper increases in females compared to males. Although our study did not include data regarding the participants’ eating habits, previous studies reported deterioration of metabolic parameters during the social distancing measures associated with unhealthy dietary habits [30]. A study in Italy showed the score of depression, anxiety, and stress was more pronounced in female than in male during the pandemic lockdown [31]. This exacerbation of mental health may have adversely affected both physical activity and eating behaviors.

This study had several limitations. First, selection bias could not be ruled out. The participants were limited to health checkup examinees at a single university hospital. They were individuals who visited the hospital regularly for health checkups even during the pandemic; thus, compared with the general population, they were likely to be more concerned about their health, live a relatively healthy lifestyle, and have a higher socioeconomic status. Previous studies have shown a greater increase in the prevalence of metabolic syndrome in these high socioeconomic status populations than in the general population [32,33], which is consistent with our study’s findings. Further studies with data on general populations considering various confounders that can affect the prevalence of metabolic syndrome are warranted. Second, this study was conducted at a single center in Seoul, the capital city of Korea. Therefore, our results cannot be generalized to the Korean population. Third, although we collected data on alcohol consumption and physical activity via self-reported questionnaires, dietary data could not be obtained. Moreover, self-reported data are prone to a recall bias. Fourth, data on insulin resistance markers were lacking. Thus, our findings may be insufficient for drawing a definitive conclusion regarding the deterioration of insulin resistance during the COVID-19 pandemic. Finally, we did not consider the history of COVID-19 among the participants. Studies have shown that COVID-19 is associated with deterioration in glucose and lipid metabolism [34]. Therefore, the data from participants who were affected by the disease before their health checkups may not accurately reflect the impact of social distancing measures.

Conversely, the main advantage of this study is its longitudinal retrospective cohort design and use of paired data for investigating the impact of COVID-19-related social distancing measures and lockdowns. We analyzed data recorded continuously from 4 years before the pandemic until the Korean government abolished its vaccine pass policy. Thus, we maximized the inclusion period from the implementation of high-level social distancing to its abolishment. Furthermore, we obtained consistent results for both subgroups in the present study and confirmed the results of previous retrospective cohort studies with greater reliability [17,18].

Metabolic syndrome, its components, and fat distribution worsened significantly after COVID-19-related social distancing, despite no significant changes in BMI. The acute increase in metabolic syndrome prevalence between 2019 and 2020 highlights the impact of social distancing. Further studies in the post-pandemic period are required to observe the long-term impact of social lockdowns on metabolic parameters.

## Figures and Tables

**Figure f6-epih-45-e2023081:**
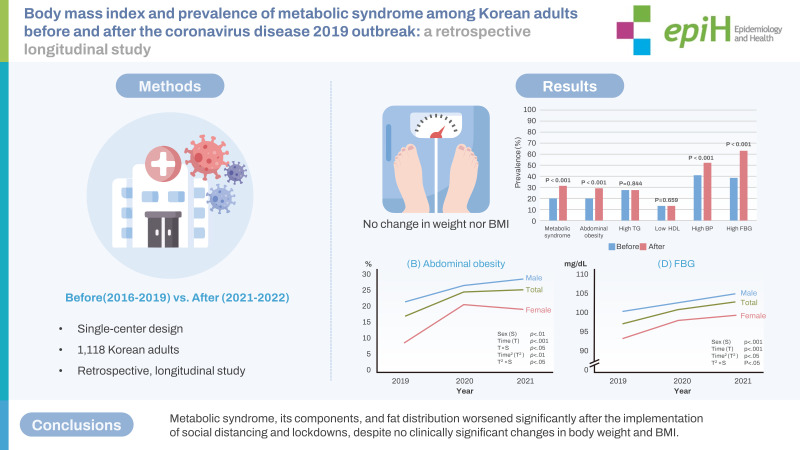


**Table 1.** Comparison of participant characteristics before and during the COVID-19 pandemic (n=1,118)

Characteristics		Before (2016-2019)	During (2021-2022)	Δ^1^	Effect size^2^	p-value^3^
Age (yr)		48.3±9.8	50.6±9.8	2.3	2.11	-
Sex, male		699 (62.5)	699 (62.5)	-	-	-
Past history						
	Hypertension	196 (17.5)	253 (22.6)	57 (5.1)	0.48	<0.001
	Dyslipidemia	94 (8.4)	168 (15.0)	74 (6.6)	0.49	<0.001
	Diabetes mellitus	57 (5.1)	86 (7.7)	29 (2.6)	0.50	<0.001
Smoking						<0.001
	Yes	219 (19.6)	173 (15.5)	46 (4.1)	-	
	No	899 (80.4)	945 (84.5)	46 (4.1)	-	
Drinking (g/wk)		78.5±24.9	81.8±21.3	3.3	0.03	0.309
Activity (METs∙min/wk)		1,831.4±1,947.6	1,500.1±1,698.7	331.3	0.16	<0.001
Height (cm)		168.1±8.1	167.8±8.2	0.3	0.20	<0.001
Weight (kg)		67.7±12.5	67.4±12.7	0.3	0.09	0.002
BMI (kg/m^2^)		23.8±3.2	23.8±3.2	0.0	0.04	0.197
Body fat (%)		26.0±6.3	26.8±6.6	0.8	0.26	<0.001
Total cholesterol (mg/dL)		194.3±36.3	197.9±40.0	3.6	0.11	<0.001
LDL-C (mg/dL)		116.5±27.9	122.2±35.4	5.7	0.21	<0.001
Components of metabolic syndrome						
	WC (cm)	79.8±9.5	81.9±10.1	2.1	0.36	<0.001
	Systolic BP (mmHg)	122.1±16.3	126.3±15.7	4.2	0.28	<0.001
	Diastolic BP (mmHg)	74.2±11.3	77.8±11.3	3.6	0.34	<0.001
	FBG (mg/dL)	99.4±17.7	106.8±19.5	7.4	0.51	<0.001
	Triglyceride (mg/dL)	131.0±98.3	131.7±100.9	0.7	0.01	0.799
	HDL-C (mg/dL)	55.4±12.6	56.5±14.0	1.1	0.13	<0.001

Values are presented as number (%) or mean±standard deviation. COVID-19, coronavirus disease 2019; MET, metabolic equivalent of task; BMI, body mass index; LDL-C, low-density lipoprotein cholesterol; WC, waist circumference; BP, blood pressure; FBG, fasting blood glucose; HDL-C, high-density lipoprotein cholesterol.

1 Difference between during and before the COVID-19 pandemic.

2 Effect size calculated with Cohen’s g for categorical variables and Cohen’s d for continuous variables.

3 Calculated with the McNemar test for categorical variables and the paired t-test for continuous variables.

## SUPPLEMENTARY MATERIALS

Supplement Material 1. Trend analysis results under the framework of the Generalized Estimating Equations (n_1_ = 400)

Supplement Material 2. Trend analysis results under the framework of the General Linear Model (n_2_ = 276)
