# Supplementary material for: Body mass index and prevalence of metabolic syndrome among Korean adults before and after the COVID-19 outbreak: a retrospective longitudinal study
Source: Epidemiol Health. 2023 Aug 29;45:e2023081. doi: 10.4178/epih.e2023081 (PMC10728615; doi:10.4178/epih.e2023081)
Supplement: Supplement Material 1. — Trend analysis results under the framework of the Generalized Estimating Equations (n1 = 400) [file epih-45-e2023081-Supplementary-1.docx]

Supplementary Material 1. Trend analysis results under the framework of the Generalized Estimating Equations (*n_1_* = 400)

|  |  | Yearly percent (%) of symptom | | |  |  |
| --- | --- | --- | --- | --- | --- | --- |
| Variable | Sex | 2019 | 2020 | 2021 | Effect | p-value |
| Metabolic syndrome | Male | 68(26.4) | 81(31.4) | 93(36.1) | S | 0.0010 |
|  | Female | 17(12.0) | 22(15.5) | 24(16.9) | T | 0.3172 |
|  |  |  |  |  | T$\times$S | 0.7747 |
|  |  |  |  |  | T^2^ | 0.6005 |
|  |  |  |  |  | T^2^$\times$S | 0.7156 |
| Abdominal obesity | Male | 55(21.3) | 68(26.4) | 73(28.3) | S | 0.0026 |
|  | Female | 13(9.2) | 29(20.4) | 27(19.0) | T | 0.0005 |
|  |  |  |  |  | T$\times$S | 0.0247 |
|  |  |  |  |  | T^2^ | 0.0051 |
|  |  |  |  |  | T^2^$\times$S | 0.0491 |
| High TG | Male | 103(39.9) | 104(40.3) | 94(36.4) | S | <.0001 |
|  | Female | 14(9.9) | 22(15.5) | 12(8.5) | T | 0.0371 |
|  |  |  |  |  | T$\times$S | 0.0804 |
|  |  |  |  |  | T^2^ | 0.0152 |
|  |  |  |  |  | T^2^$\times$S | 0.0587 |
| Low HDL-C | Male | 18(7.0) | 24(9.3) | 28(10.9) | S | 0.0136 |
|  | Female | 21(14.8) | 22(15.5) | 23(16.2) | T | 0.8393 |
|  |  |  |  |  | T$\times$S | 0.5491 |
|  |  |  |  |  | T^2^ | 0.9937 |
|  |  |  |  |  | T^2^$\times$S | 0.7751 |
| High BP | Male | 123(47.7) | 127(49.2) | 148(57.4) | S | <.0001 |
|  | Female | 26(18.3) | 30(21.1) | 44(31.0) | T | 0.9782 |
|  |  |  |  |  | T$\times$S | 0.8329 |
|  |  |  |  |  | T^2^ | 0.3059 |
|  |  |  |  |  | T^2^$\times$S | 0.8456 |
| High FBG | Male | 142(55.0) | 167(64.7) | 188(72.9) | S | <.0001 |
|  | Female | 36(25.4) | 61(43.0) | 70(49.3) | T | 0.0012 |
|  |  |  |  |  | T$\times$S | 0.1112 |
|  |  |  |  |  | T^2^ | 0.0562 |
|  |  |  |  |  | T^2^$\times$S | 0.1595 |

*Note.* S = Sex, T = Linear trend, T$\times$S = Interaction between linear trend and sex, T^2^ = Quadratic trend, T^2^$\times$S = Interaction between quadratic trend and sex

TG, triglyceride; HDL-C, high-density lipoprotein cholesterol; BP, blood pressure; FBG, fasting blood glucose
