# Supplementary material for: Body mass index and prevalence of metabolic syndrome among Korean adults before and after the COVID-19 outbreak: a retrospective longitudinal study
Source: Epidemiol Health. 2023 Aug 29;45:e2023081. doi: 10.4178/epih.e2023081 (PMC10728615; doi:10.4178/epih.e2023081)
Supplement: Supplement Material 2. — Trend analysis results under the framework of the General Linear Model (n2 = 276) [file epih-45-e2023081-Supplementary-2.docx]

Supplementary Material 2. Trend analysis results under the framework of the General Linear Model (*n_2_* = 276)

|  |  | Yearly Mean ± Standard Deviation | | |  |  |
| --- | --- | --- | --- | --- | --- | --- |
| Variable | Sex | 2019 | 2020 | 2021 | Effect | p-value |
| WC | Male | 82.3±7.4 | 83.2±8.0 | 84.4±7.6 | S | <.0001 |
|  | Female | 69.9±6.0 | 72.1±7.8 | 72.3±7.7 | T | <.0001 |
|  |  |  |  |  | T$\times$S | 0.5815 |
|  |  |  |  |  | T^2^ | 0.0854 |
|  |  |  |  |  | T^2^$\times$S | 0.0343 |
| HDL-C | Male | 54.2±10.2 | 54.6±12.0 | 53.1±10.5 | S | <.0001 |
|  | Female | 61.4±12.9 | 64.0±15.8 | 63.0±14.1 | T | 0.6490 |
|  |  |  |  |  | T$\times$S | 0.0091 |
|  |  |  |  |  | T^2^ | 0.0038 |
|  |  |  |  |  | T^2^$\times$S | 0.3148 |
| TG^a^ | Male | 4.8±0.5 | 4.8±0.5 | 4.8±0.5 | S | <.0001 |
|  | Female | 4.4±0.4 | 4.5±0.4 | 4.5±0.4 | T | 0.1781 |
|  |  |  |  |  | T$\times$S | 0.9883 |
|  |  |  |  |  | T^2^ | 0.0366 |
|  |  |  |  |  | T^2^$\times$S | 0.7541 |
| FBG | Male | 100.9±15.0 | 103.4±14.1 | 105.7±17.8 | S | <.0001 |
|  | Female | 94.1±11.1 | 98.8±10.1 | 100.1±11.8 | T | <.0001 |
|  |  |  |  |  | T$\times$S | 0.2585 |
|  |  |  |  |  | T^2^ | 0.0128 |
|  |  |  |  |  | T^2^$\times$S | 0.0457 |
| Systolic BP | Male | 121.3±14.4 | 122.2±13.2 | 126.1±14.7 | S | <.0001 |
|  | Female | 111.1±11.9 | 114.9±13.1 | 118.0±14.2 | T | <.0001 |
|  |  |  |  |  | T$\times$S | 0.2100 |
|  |  |  |  |  | T^2^ | 0.3291 |
|  |  |  |  |  | T^2^$\times$S | 0.1319 |
| Diastolic BP | Male | 73.3±10.6 | 74.8±10.3 | 77.6±11.3 | S | <.0001 |
|  | Female | 67.1±9.2 | 70.1±9.5 | 72.3±11.2 | T | <.0001 |
|  |  |  |  |  | T$\times$S | 0.4895 |
|  |  |  |  |  | T^2^ | 0.8389 |
|  |  |  |  |  | T^2^$\times$S | 0.2854 |

^a^ Log transformation due to substantial skewness

*Note.* S = Sex, T = Linear trend, T$\times$S = Interaction between linear trend and sex, T^2^ = Quadratic trend, T^2^$\times$S = Interaction between quadratic trend and sex

WC, waist circumference; HDL-C, high-density lipoprotein cholesterol; FBG, fasting blood glucose; BP, blood pressure
